# Comparative analysis of optical properties in amniotic membrane products

**DOI:** 10.1007/s10792-025-03805-x

**Published:** 2025-11-13

**Authors:** Bryanna J. Lee, Jeffrey Tsao, Natalie A. Afshari

**Affiliations:** Shiley Eye Institute, Viterbi Family Department of Ophthalmology, University of California San Diego, 9415 Campus Point Drive, La Jolla, San Diego, CA 92037 USA

**Keywords:** Amniotic membrane, Corneal surgery, Optical quality, Blur index, Vision

## Abstract

**Purpose:**

Compare optical properties of amniotic membranes (AM) using a modified photographic-based method.

**Methods:**

Optical measures of various AMs (Prokera® Plus, Prokera® Slim, Amniograft®, Ambio2®, Ambio5®) and a Collagen Shield were quantified by examining standardized band pattern images through the AMs. Blur Index (BI) was calculated by comparing standard deviations of gray values, while transparency ratio (TR) was determined by comparing mean peak gray values of white bars viewed through the AMs.

**Results:**

BIs ranged from 0.97% (Ambio2®) to 22.58% (Ambio5®), with 0% signifying minimal blurring. TRs ranged between 0.906 to 1.018, with greater values signifying more brightness. BI and TR showed an inverse correlation (r = − 0.954, 95% CI − 0.984, − 0.871). Thicker AMs generally demonstrated higher BIs. Cross-linking effects were unpredictable.

**Conclusion:**

TR inversely correlated with BI and thicker AMs generally exhibited higher BIs. This method offers a rapid, cost-effective approach to comparing AM optical qualities, providing insights into patient vision.

## Introduction

Corneal pathology represents a leading cause of blindness worldwide [1]. The human cornea, a transparent tissue responsible for the majority of the eye’s refractive power, plays a crucial role in focusing light onto the retina. Amniotic membrane (AM), derived from the innermost layer of the placenta, possesses unique anti-inflammatory, anti-scarring, and anti-angiogenic properties that promote corneal healing and regeneration [2–4]. AM serves as a scaffold for epithelial cell growth and aids in the reconstruction of the ocular surface in conditions such as persistent epithelial defects, chemical or thermal burns, limbal stem cell deficiency, and microbial keratitis [5].

The structural similarities between AM and the cornea, including the absence of blood vessels, the arrangement of collagen fibrils, and the presence of a basement membrane, make it an ideal candidate for corneal tissue engineering [6]. However, the optical properties of AM, particularly its transparency and potential for visual blurring, are critical factors that warrant careful consideration in clinical applications.

The transparency of AM can vary significantly depending on its source, preparation method, and preservation technique. Factors such as the region of the membrane used, the removal of epithelial cells and spongy layer [6], and the application of techniques like freeze-drying [7, 8] or chemical cross-linking [9] can all influence the optical clarity and mechanical properties of AM.

While several methods exist for evaluating the optical properties of AM, a photographic-based method, previously described by Gonzales et al. [10], offers an objective and quantitative measure of functional optical blurring and transparency, based on simple cost-effective technology. This technique involves analyzing photographic images of a standardized band pattern taken through the sample membranes, with a fixed-width analysis at each black and white transition of photographic images.

Our study builds upon the photographic-based method by analyzing a sample-wide section of each membrane instead of a fixed-width analysis at each black and white transition. This approach potentially allows for more comprehensive and accurate representations of characteristics across the entire sample. We utilize this modified method to compare the optical qualities of different commercially available amniotic membranes, focusing on the effects of thickness and cross-linking on optical quality. Cross-linking has been shown to enhance AM biomechanical strength, stability and resistance to enzymatic degradation while reducing the rate of biodegradation [11, 12].

Importantly, this revised method provides insight into patient vision when AM is applied, offering a quantitative measure of the potential blurriness experienced. By providing an objective, accessible, and comprehensive analysis of AM optical properties, our approach aims to assist clinicians in product selection and improve understanding of how these membranes may affect patient vision during treatment.

## Methods

This study evaluated distinct types of amniotic membranes (AM): three cryopreserved (Prokera® Plus, Prokera® Slim, Amniograft®) and two dehydrated (Ambio2®, Ambio5®). A Collagen Shield served as a control. Two samples of each AM type were analyzed, with the exception of dehydrated samples, for which three were examined (total n = 13).

Transparency and image blurring measurements were obtained by analyzing photographic images of a standardized band pattern taken through the AMs. A black and white stripe pattern was displayed on a laptop screen as described by Gonzales et al. [10]. Photographic images of the standardized black and white band pattern (10 alternating vertical bars, each 3 mm wide) was displayed on a 13-inch MacBook Pro liquid crystal display screen with 100% brightness. A petri dish with each AM sample was placed over this pattern, and baseline comparator measures were made using an identical empty petri dish. The distance between the computer screen and the camera objective was fixed at 32 cm, and images were captured using manual exposure settings to ensure consistent comparison. Each AM sample was mounted flat on a clear petri dish positioned in front of the display.

**Fig. 1:**
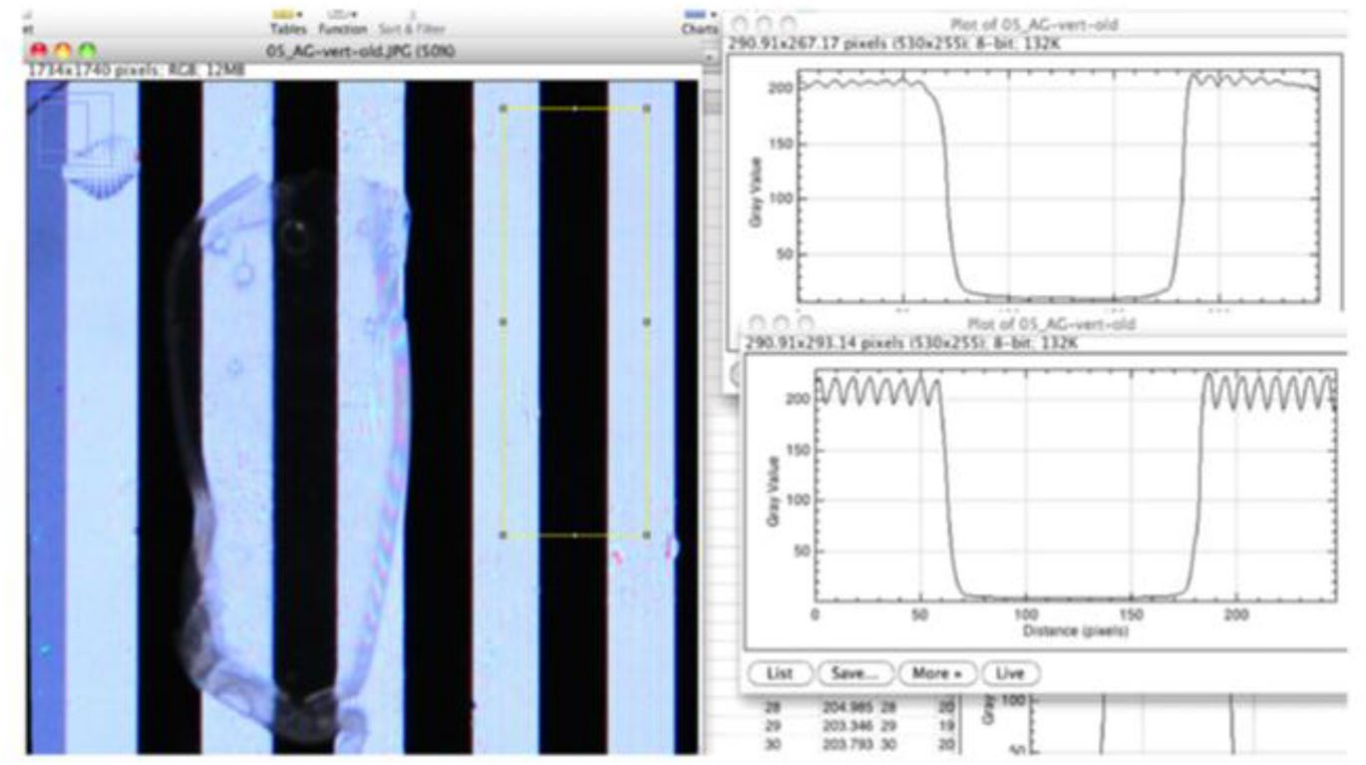
Screenshot of image analysis with gray value graphs comparing Amniograft® (upper) to Petri dish (lower). The slopes surrounding each trough are steeper for the latter, indicating a sharper transition from black to white, a proxy for visual sharpness

Images were imported and analyzed with Image J software. For each sample, gray value intensity plots were generated across the full width of the membrane. The blur index (BI) was calculated as the ratio of standard deviations of gray values observed through the AM compared to the underlying petri dish ($$BI = 1 - \frac{{SD_{AM profile} }}{{SD_{empty petri dish} }}$$), where the SD_AM profile_ is the standard deviation obtained for a profile with the amniotic membrane placed on top of the petri dish, and the SD_empty petri dish_ is the standard deviation obtained for a profile with no sample placed on top of the petri dish. The transparency ratio (TR) was calculated to estimate transmittance ($$TR = \frac{{MAX_{AM} }}{{MAX_{petri dish} }}$$), where the MAX_AM_ is the MAX value of each AM and the MAX_petri dish_ is the MAX value obtained in the petri dish control. Our method differed from Gonzales et al. in that our analysis encompassed a sample-wide section of each membrane rather than fixed 354 µm segments at each black/white transition (Fig. 1).

To examine the effects of cross-linking, a sample of each AM type was treated with a 0.1% riboflavin solution and exposed to ultraviolet light. Optical measurements were then repeated on cross-linked samples.

Statistical analysis was performed using GraphPad Prism. A *p*-value < 0.05 was considered statistically significant.

## Results

**Fig. 2:**
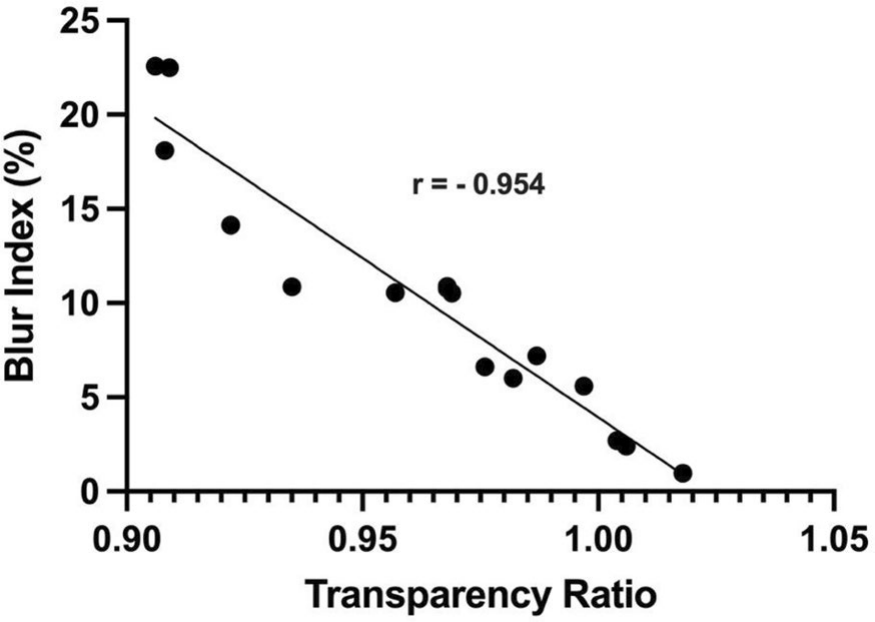
Scatterplot of blur index versus transparency values

The blur indices among all tested AMs ranged from 0.97% (Ambio2®) to 22.58% (Ambio5®), with 0% signifying minimal blurring. Each brand of AM tended to have blur index values that somewhat clustered together; however there was strong intra-brand variability. Prokera® Slim exhibited the lowest mean blur index at 4.8% ± 3.3%, while Ambio5® exhibited the largest mean blur index at 18.6% ± 6.6%. The transparency ratios varied between 0.906 and 1.018, with higher values representing greater brightness. A strong inverse correlation was observed between blur index and transparency ratio (r = − 0.954, 95%; CI − 0.984, − 0.871). (Fig. 2). A table of all blur index and transparency ratio values are shown in Table 1.

**Table 1:** Blur indices and transparency ratios of various amniotic membranes

Membrane type	Preservation method	Thickness (µm)*	Blur index (%)	Transparency ratio
Prokera® Plus	Cryopreserved	100–150	10.53	0.969
Prokera® Plus	Cryopreserved	100–150	10.55	0.957
Prokera® Slim	Cryopreserved	50–70	7.20	0.987
Prokera® Slim	Cryopreserved	50–70	2.41	1.006
Amniograft®	Cryopreserved	50–100	10.88	0.968
Amniograft®	Cryopreserved	50–100	2.69	1.004
Ambio2®	Dehydrated	50–100	6.00	0.982
Ambio2®	Dehydrated	50–100	0.97	1.018
Ambio2®	Dehydrated	50–100	10.87	0.935
Ambio5®	Dehydrated	100–200	22.58	0.906
Ambio5®	Dehydrated	100–200	10.78	0.968
Ambio5®	Dehydrated	100–200	22.48	0.909
Collagen Shield	N/A	100	5.60	0.997

*Reference values from BioTissue (BioTissue, 2025), IOP Opthalmics (Panfundus, 2025), and Kestrel Opthalmics (Opthalmics, 2025) websites

**Fig. 3:**
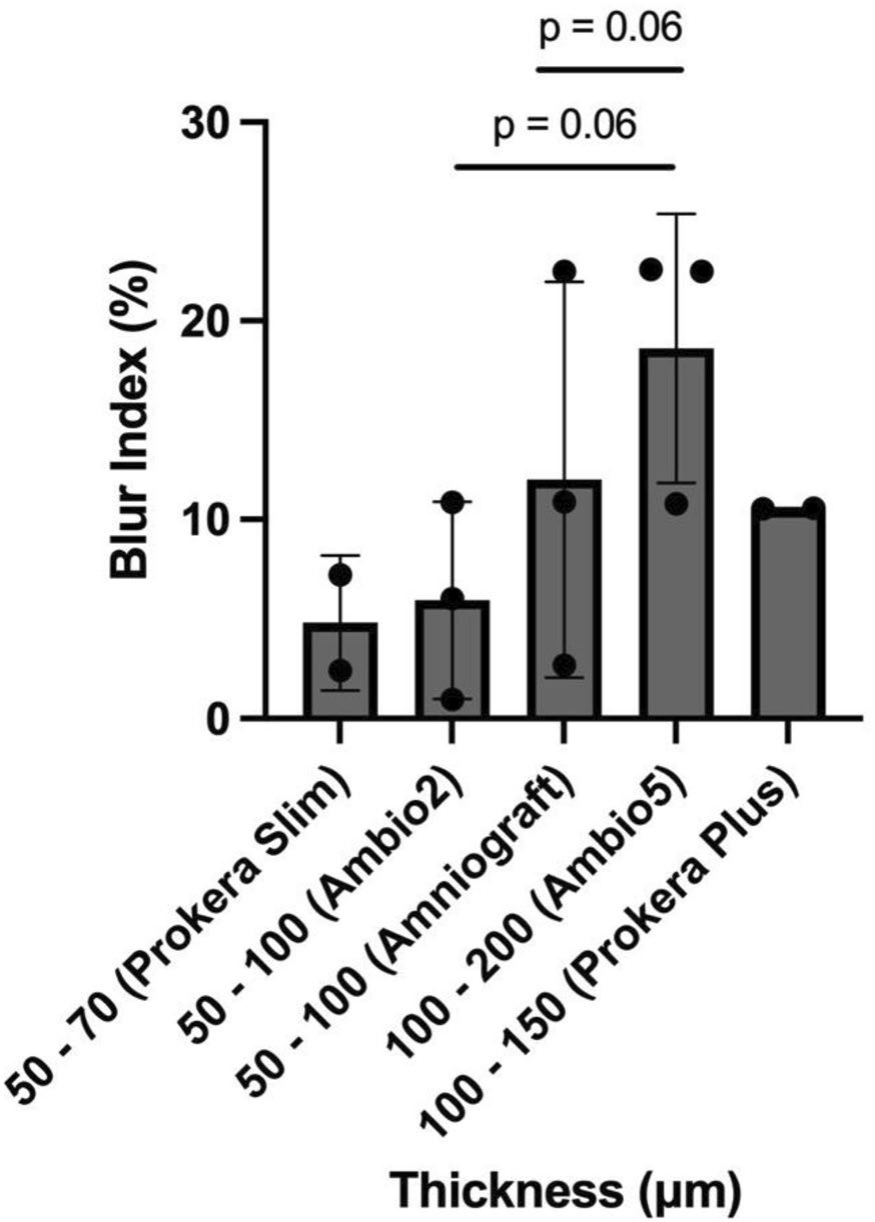
Thickness of amniotic membranes versus blur indices

Thicker AMs tended to have higher blur indices than thinner ones. The increase in blur index from Ambio2® (50–100 μm) and Prokera® Slim (50–70 μm) to Ambio5® (110–200 μm) approached significance (*p* = 0.06) (Fig. 3)

Cross-linking treatment resulted in varied effects on the optical properties of the AMs (range: + 32.1 to − 36.0%). In general, cross-linking reduced blur index for four out of six membranes, however none were statistically significant. Cross-linking increased the blur index of Collagen Shield and Prokera® Plus by 11.3% and 23.1%, respectively. The effects on transparency values were less pronounced, ranging from a 5% reduction to a 3% increase, with no statistical significance observed.

## Discussion

The modified method used in this study provides a reliable quantification of AM optical properties based on a simple and inexpensive methodology. The strong inverse correlation observed between blur index and transparency ratio (r = − 0.954, 95% CI − 0.984, − 0.871) agrees with previously shown data and validates the technique’s consistency while offering comprehensive sample-wide analysis [7, 10].

Our study revealed significant variations in optical properties among different AM products, with blur indices ranging from 0.97 to 22.58% and TRs between 0.906 and 1.018. While blur index values tended to cluster within brands, notable intra-brand variability was exhibited. The inverse relationship between blur index and transparency ratio indicates that increased blurring correlates with decreased light transmission, underscoring the importance of considering optical characteristics when selecting AMs for ocular surface reconstruction, particularly in cases where visual outcomes are critical.

Evaluating the thickness of AMs and the blur index allowed us to compare the optical properties between the AMs. Prokera® Plus, designed for enhanced structural support and durability, was the thickest AM examined. Ambio5®, a multi-layered, dehydrated AM, was thicker than the single-layered, dehydrated Ambio2®. Amniograft®, a cryopreserved amniotic membrane, falls in between Ambio2® and Ambio5® in terms of thickness. Prokera® Slim, aimed at providing patient comfort, was the thinnest. In our study, thicker AMs generally had higher blur indices compared to thinner AMs, and therefore correlated with lower transparency. The observed range in blur indices likely stems from the inherent heterogeneity of human AMs, which consist of distinct regions such as placental, reflected, and umbilical amnion. For example, the placental region of the amniotic membrane is generally thicker and less transparent compared to the reflected region [6, 14]. While the specific origin of the AM used in commercial products is often not disclosed, this factor, along with variations in processing methods, likely contributes to the differences in thickness and blur index. It is important to note that these optical properties do not necessarily reflect the respective AMs’ healing capabilities. Therefore, the selection of AMs for clinical use should consider both optical characteristics and therapeutic potential, depending on the specific requirements for each use.

Cross-linking has been shown to improve light transmittance of AMs [9, 13], however, excessive UV exposure can lead to the scission of collagen chains, potentially reducing transparency. The effects of our cross-linking treatment showed considerable variability, with an observed range of + 32.1 to − 36.0% change in blur index. While these results suggest that cross-linking alters the structural and functional properties of AMs, there is a need for more controlled studies with larger sample sizes to establish definitive relationships between cross-linking and optical properties.

In addition to its utility for research comparisons, this photographic-based method could also serve as a standardized release criterion for amniotic membrane manufacturers. Incorporating routine assessment of blur index and transparency ratio during processing would provide objective, reproducible data on optical quality that could be included with product specifications. Such information would enable clinicians to make more informed decisions, selecting membranes with higher transparency for cases involving the visual axis and reserving less transparent membranes for applications where optical clarity is less critical, such as tectonic support or peripheral coverage. While further validation and larger-scale studies are needed to establish regulatory acceptance, this approach highlights the potential for our method to extend beyond laboratory evaluation into manufacturing quality control and clinical practice.

An important limitation of this study is the small sample size used for each AM product and cross-linking treatment. While our results provide insights in to the optical properties of various AM products and the effects of cross-linking, the limited number of samples may have contributed to the lack of statistical significance in some comparisons. Future studies should aim to include a greater number of samples for each AM to provide more definitive conclusions and strengthen the generalizability of the findings. Furthermore, an additional source of variability may stem from donor-to-donor differences, as commercial AMs do not disclose tissue IDs. Donor-related heterogeneity, including regional membrane thickness and collagen composition, could therefore have contributed to the variation in transparency and blur indicies observed in our study.

## Conclusion

This modified method of assessing blur index and optical qualities of AMs presents an efficient, inexpensive, and objective approach that offers insights into potential patient vision when applied. The quantitative measurements of blur index and transparency ratio provide valuable guidance for clinical product selection when considered alongside mechanical strength and healing characteristics. By offering an analysis of AM optical properties, this modified technique not only assists clinicians in product selection, but also enhances our understanding of how these membranes may affect patient vision during treatment. While this method demonstrates potential for predicting visual outcomes, more validation is required through controlled trials.

## Data Availability

No datasets were generated or analysed during the current study.

## References

[1] Whitcher JP, Srinivasan M, Upadhyay MP (2001) Corneal blindness: a global perspective. Bull World Health Organ 79(3):214–221 11285665 PMC2566379

[2] Hao Y, Ma DH, Hwang DG, Kim WS, Zhang F (2000) Identification of antiangiogenic and antiinflammatory proteins in human amniotic membrane. Cornea 19(3):348–352. 10.1097/00003226-200005000-00018 10832697 10.1097/00003226-200005000-00018

[3] Kim JS, Kim JC, Na BK, Jeong JM, Song CY (2000) Amniotic membrane patching promotes healing and inhibits proteinase activity on wound healing following acute corneal alkali burn. Exp Eye Res 70(3):329–337. 10.1006/exer.1999.0794 10712819 10.1006/exer.1999.0794

[4] Solomon A, Rosenblatt M, Monroy D, Ji Z, Pflugfelder SC, Tseng SC (2001) Suppression of interleukin 1alpha and interleukin 1beta in human limbal epithelial cells cultured on the amniotic membrane stromal matrix. Br J Ophthalmol 85(4):444–449. 10.1136/bjo.85.4.444 11264135 10.1136/bjo.85.4.444 PMC1723909

[5] Sanders FWB, Huang J, Alió del Barrio JL, Hamada S, McAlinden C (2024) Amniotic membrane transplantation: structural and biological properties, tissue preparation, application and clinical indications. Eye 38(4):668–679. 10.1038/s41433-023-02777-5 37875701 10.1038/s41433-023-02777-5 PMC10920809

[6] Deihim T, Yazdanpanah G, Niknejad H (2016) Different light transmittance of placental and reflected regions of human amniotic membrane that could be crucial for corneal tissue engineering. Cornea 35(7):997–1003. 10.1097/ico.0000000000000867 27149533 10.1097/ICO.0000000000000867

[7] Connon CJ, Doutch J, Chen B, Hopkinson A, Mehta JS, Nakamura T, Kinoshita S, Meek KM (2010) The variation in transparency of amniotic membrane used in ocular surface regeneration. Br J Ophthalmol 94(8):1057–1061. 10.1136/bjo.2008.153064 19304581 10.1136/bjo.2008.153064

[8] Jirsova K, Jones GLA (2017) Amniotic membrane in ophthalmology: properties, preparation, storage and indications for grafting-a review. Cell Tissue Bank 18(2):193–204. 10.1007/s10561-017-9618-5 28255771 10.1007/s10561-017-9618-5

[9] Tanaka Y, Kubota A, Yokokura S, Uematsu M, Shi D, Yamato M, Okano T, Quantock AJ, Nishida K (2012) Optical mechanical refinement of human amniotic membrane by dehydration and cross-linking. J Tissue Eng Regen Med 6(9):731–737. 10.1002/term.479 22489071 10.1002/term.479

[10] Gonzalez-Andrades M, Cardona Jde L, Ionescu AM, Mosse CA, Brown RA (2015) Photographic-based optical evaluation of tissues and biomaterials used for corneal surface repair: a new easy-applied method. PLoS ONE 10(11):e0142099. 10.1371/journal.pone.0142099 26566050 10.1371/journal.pone.0142099 PMC4643926

[11] Ma DH, Lai JY, Cheng HY, Tsai CC, Yeh LK (2010) Carbodiimide cross-linked amniotic membranes for cultivation of limbal epithelial cells. Biomaterials 31(25):6647–6658. 10.1016/j.biomaterials.2010.05.034 20541801 10.1016/j.biomaterials.2010.05.034

[12] Skopinska-Wisniewska J, Michalak M, Tworkiewicz J, Tyloch D, Tuszynska M, Bajek A (2023) Modification of the human amniotic membrane using different cross-linking agents as a promising tool for regenerative medicine. Materials (Basel). 10.3390/ma16206726 37895710 10.3390/ma16206726 PMC10608722

[13] Spoerl E, Wollensak G, Reber F, Pillunat L (2004) Cross-linking of human amniotic membrane by glutaraldehyde. Ophthalmic Res 36(2):71–77. 10.1159/000076884 15017101 10.1159/000076884

[14] Weidinger A, Poženel L, Wolbank S, Banerjee A (2020) Sub-regional differences of the human amniotic membrane and their potential impact on tissue regeneration application. Front Bioeng Biotechnol 8:613804. 10.3389/fbioe.2020.613804 33520964 10.3389/fbioe.2020.613804 PMC7839410

